# 2,4-Dihydroxy-3′-methoxy-4′-ethoxychalcone suppresses cell proliferation and induces apoptosis of multiple myeloma *via* the PI3K/akt/mTOR signaling pathway

**DOI:** 10.1080/13880209.2019.1662814

**Published:** 2019-09-28

**Authors:** FengChen Zhu, DianMing Jiang, MingHua Zhang, Bo Zhao

**Affiliations:** a Department of Orthopaedics, The Yongchuan Affiliated Hospital, Chongqing Medical University, Chongqing, P.R. China;; b Department of Orthopaedics, Third Affiliated Hospital, Chongqing Medical University, Chongqing, P.R. China

**Keywords:** *Caragana pruinosa* Kom., U266, cleaved-caspase-3, cleaved-caspase-9, Bcl-2, Z-VAD-FAK, IGF-1

## Abstract

**Context:** *Caragana pruinosa* Kom. (Fabaceae), a commonly used folk medicine, has been found to possess antitumor effects. However, the antiproliferative effect of 2,4-dihydroxy-3′-methoxy-4′-ethoxychalcone (DMEC) derived from *C. pruinosa* against multiple myeloma (MM) has never been investigated.

**Objective:** This study systematically evaluates the antiproliferative effect of DMEC against MM cells.

**Materials and methods:** The antiproliferative effect of DMEC (1, 2, 4, 8, 16, 32, and 64 μM) on MM cells lines, including RPMI8226, MM.1S, and U266, was examined using Cell counting kit-8 (CCK-8) assay after 24 h incubation. The proapoptotic effect of DMEC (20 μM) was determined using fluorescent microscope and flow cytometer, and its possible underlying mechanisms were further studied by using western blotting analysis.

**Results:** The half maximal inhibitory concentrations (IC_50_) of DMEC on RPMI8226, MM.1S, and U266 cells were calculated as 25.97, 18.36, and 15.02 μM, respectively. The inhibitory effect of DMEC on MM cells was related to mitochondria-mediated apoptosis *via* upregulation of the cleaved-caspase-3 (C-3), cleaved-caspase-9 (C-9), Bad, and cytochrome C (Cyto C), but downregulation of the Bcl-2 and poly ADP-ribose polymerase (PARP). Furthermore, DMEC (5, 10, and 20 μM) reduced the expression of phosphatidylinositol-3-kinase (PI3K), phosphorylated (p)-protein kinase B (Akt), and p-mammalian target of rapamycin (p-mTOR), which were further evidenced by pretreatment with IGF-1, a PI3K activator.

**Conclusion:** Collectively, our results indicate that the DMEC could be treated as a new candidate for treatment of multiple myeloma in the future. Also, an *in vivo* study is warranted in the future.

## Introduction

Multiple myeloma (MM), also termed as myeloma or plasma cell myeloma, is a type of malignancy characterized by uncontrolled increase of plasma cells within the bone marrow (Rajkumar 2012; Chavda and Yong 2017). If untreated, patients with MM have a survival time of only six months; however, the survival time may be longer than 3 years when drug therapy is received (Shu et al. 2016). Thus, MM is currently considered as an incurable disease that urgently requires novel agents to alleviate the symptoms, such as bone pain, infection, and hypercalcemia (Toocheck and Pinkhas 2018). Currently, the available agents, including bortezomib, thalidomide, and lenalidomide, have been used as part of the frontline treatment for MM. However, long-term administration of these synthetic drugs commonly cause fatigue, nausea, diarrhea, loss of appetite, and some other side effects (Durusu et al. 2017; Moreau et al. 2013). Therefore, it is urgent for us to develop more effective and reliable medications with few side effects and toxicity for curing MM.

**Figure 1.**
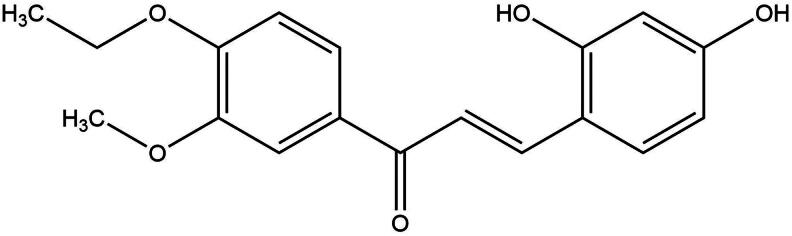
Chemical structure of 2,4-dihydroxy-3′-methoxy-4′-ethoxychalcone (DMEC).

Increasing evidence demonstrated that natural products extracted from plants/herbs are potential resources for discovering candidate drugs for treating cancers/tumors (Zhou et al. 2005; Peng et al. 2019). *Caragana pruinosa* Kom. (Fabaceae) is a small deciduous shrub mainly distributed in Xinjiang province of China and some Middle Asian countries of Kazakhstan, and Kyrgyzstan (Wu et al. 2018; Zheng et al. 2016). It has been revealed that *C. pruinosa* is a reliable folk medicine with anti-inflammatory, antiproliferative, antimicrobial, and analgesic effects (Meng et al. 2009; Niu et al. 2014). Furthermore, phytochemical investigations found that *C. pruinosa* possess abundant chemical components, including flavonoids, terpenoids, stilbenoids, etc. (Luo et al. 2001; Shi et al. 2003; Sun et al. 2004). Previous studies have suggested that the majority of flavonoid compounds isolated from the genus *Caragana* exhibited inhibitory effects against breast, cervix, and liver cancer (Qiu and Piao 2012; Su et al. 2017). As part of our continuing efforts focused on compounds obtained from *C. pruinosa*, we found that 2,4-dihydroxy-3′-methoxy-4′-ethoxychalcone (DMEC; Figure 1), one of the flavonoid constituents, notably inhibited proliferation of the MM cell line U266. Thus, the current study was designed to systematically evaluate the antiproliferative effect of DMEC against MM cells and further investigated its possible molecular mechanisms.

## Materials and methods

### Chemicals

DMEC was obtained from Shanghai Yuanyebio Tech. Ltd. Co. (Shanghai, China). Cell counting kit-8 (CCK-8), BCA protein assay kit, and goat anti-rabbit horseradish peroxidase (HRP)-conjugated secondary antibodies (catalog no. A0208) were obtained from Beyotime Institute of Biotechnology (Haimen, China). Hoechst 33324 was obtained from Sigma Co. (Shanghai, China). IP cell lysis buffer containing 20 mM Tris (pH 7.5), 150 mM NaCl, 1% Triton X-100, sodium pyrophosphate, β-glycerophosphate, EDTA, Na_3_VO_4_ and leupeptin were obtained from Sangon Biotech (Shanghai, China). Annexin V-FITC/PI kit was obtained from BD Biosciences (CA, USA). Cleaved-caspase-3 (C-3) (dilution 1:1000), cleaved-caspase-9 (C-9) (dilution 1:1000) antibodies were obtained from Cell Signaling Technology (MA, USA). Bad (dilution 1:3000), Bcl-2 (dilution 1:800), phosphorylated (p)-Akt (dilution 1:1000), p-PI3K (dilution 1:1000), p-mTOR (dilution 1:1000), Akt (dilution 1:500), PI3K (dilution 1:100), mTOR (dilution 1:2000), Cytochrome C (Cyto C; dilution 1:2000), poly ADP-ribose polymerase (PARP; dilution 1:1000), and GAPDH (dilution 1:1000) antibodies, and Z-LEHD-FMK inhibitor were obtained from Abcam Biotechnology (Cambridge, UK). Other chemicals used in the current study were of analytical reagent grade.

### Cell culture

Human MM cell lines RPMI8226, MM.1S, and U266 were purchased from American Type Culture Collection (ATCC, Manassas, VA, USA). The three MM cell lines were cultured in RPMI-1640 medium, containing 10% (v/v) fetal bovine serum (FBS), 1% (v/v) penicillin, 1% (v/v) l-glutamine and 100 μg/mL streptomycin, in a 5% CO_2_ humidified atmosphere at 37 °C.

### CCK-8 assay

Cell viability of RPMI8226, MM.1S, and U266 were carried out by using CCK-8 assay. Briefly, cells were seeded in 96-well plate at a density of 8 × 10^3^ cells/well. After 24 h incubation, the cells lines were exposed to DMEC at series concentrations (1, 2, 4, 8, 16, 32, and 64 μM) for an additional 24 h. Subsequently, CCK-8 solution was added to determine cell proliferation inhibition (%). The optical density (OD) was measured using a microplate reader (Bio-Rad, CA, USA) at 450 nm, and the IC_50_ values of DMEC on RPMI8226, MM.1S, and U266 cells were obtained. The inhibition rate was calculated according to the following formula: (OD_control_−OD_treatment_)/OD_control_ × 100%.

To evaluate the potential toxicity of DMEC, spleen lymphocytes removed from ICR mice was employed. The animal protocols were approved with Animal Experimentation Ethics Committee of Chongqing Medical University (Approval No. An-2018-010301#). The mouse spleen was transferred to culture dish, grinded with Hanks’ buffer, and then filtered with 200 mesh nylon mesh. Following with centrifuging at 12,000 *g* for 10 min at 4 °C, the lower cells were collected as the spleen lymphocytes. The toxicity of DMEC on spleen lymphocytes was determined by using CCK-8 assay. Briefly, spleen lymphocytes were seeded into 96-well plate at density of 2 × 10^6^ cells/well. After incubation for 24 h, cells lines were exposed to DMEC at concentrations of 2, 5, 10, 20, 40, 80, and 160 μM for another 36 h. The subsequent experimental methods are consistent with the above.

### Nuclear staining with Hoechst 33324

The pro-apoptotic effect of DMEC on MM cells was examined by Hoechst 33324 staining, as previously described (Saha et al. 2017). Briefly, U266 cells (1 × 10^5^ cells/well) were seeded into 6-well plates and then treated with DMEC at dose of 20 μM for 24 h. Subsequently, cells were stained with 1 μg/mL Hoechst 33324 for 10 min in the dark at 37 °C. Finally, changes in the cell nucleus were visualized and photographed under a fluorescent microscope (Olympus Corporation, Tokyo, Japan).

### Apoptosis assay by flow cytometry

U266 cells were seeded into 6-well culture plates and treated with DMEC at 20 μM for 24 h. After collection, cells were incubated with 10 μL FITC-conjugated Annexin V in binding buffer for 15 min in the dark. Then, 10 μL propidium iodide (PI) was also added to the cell suspension, and apoptotic cells were examined using a flow cytometer assay on a FACS calibur flow cytometer (BD Biosciences, CA, USA).

### Western blotting analysis

Cells were collected and homogenized with IP cell lysis buffer, and the homogenate was centrifuged at 12,000 *g* for 10 min to extract the total protein. After determination of protein concentration using a BCA protein assay kit, 30 μg proteins were separated by sodium dodecylsulfate-polyacrylamide gel electrophoresis (SDS-PAGE), which were then blotted onto a PVDF membrane. Following blocking with 5% nonfat milk, proteins on the PVDF membrane were probed with primary monoclonal antibodies overnight at 4 °C with agitation. The PVDF membrane was then incubated with HRP-conjugated secondary antibodies for 2 h at room temperature. Finally, the target protein bands were visualized by chemiluminescence method using a commercial ECL kit (Beyotime Institute of Biotechnology, Haimen, China). The OD value was analyzed using ImageJ software from the National Institutes of Health (Bethesda, MD, USA). GAPDH was used as the internal reference for normalization.

### Statistical analysis

Data are expressed as the mean ± standard deviation. All statistical analysis was performed using one-way ANOVA followed by a LSD test for multiple comparisons by SPSS 19.0 software (SPSS, Inc., Chicago, USA). *p* < 0.05 was considered as statistically significant difference.

## Results

### Inhibitory effect of DMEC on multiple myeloma cell lines

**Figure 2.**
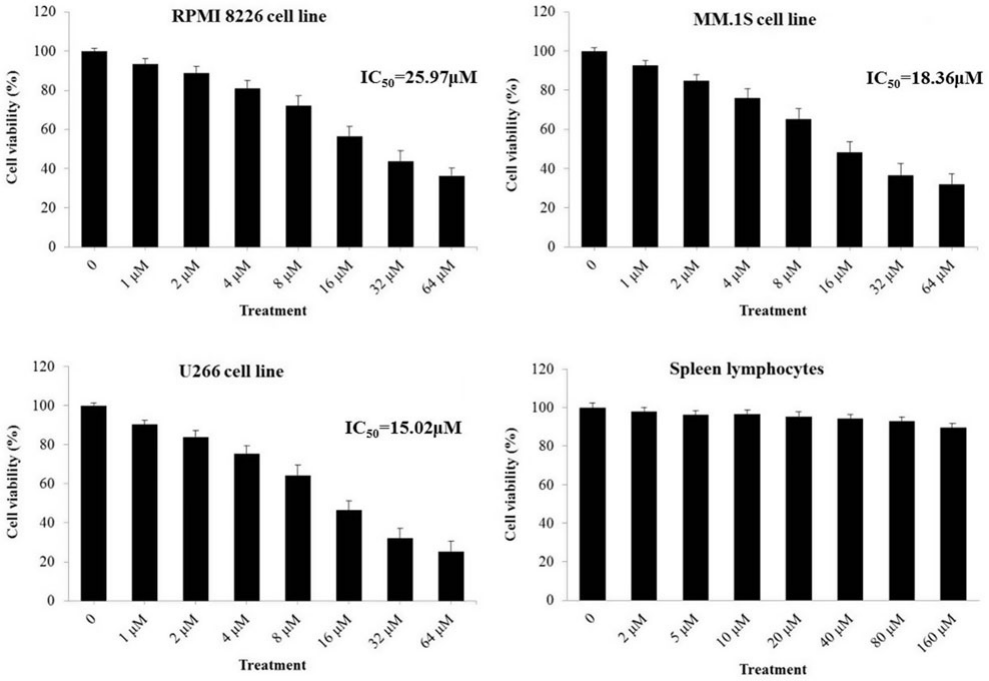
Cell viability of DMEC on MM cells lines RPMI8226, MM.1S, and U266, and normal spleen lymphocytes. Cells were treated with DMEC (1–160 μM) for 24 h. Subsequently, CCK-8 assay was used to determine cell proliferation inhibition (%). Data are presented as the mean ± standard deviation (*n* = 4).

As presented in Figure 2, DMEC treatment exhibited significantly inhibitory effect on proliferation of the three MM cell lines of RPMI8226, MM.1S, and U266 with IC_50_ values of 25.97, 18.36, and 15.02 μM, respectively, in a concentration-dependent manner (1, 2, 4, 8, 16, 32, and 64 μM). The U266 cell line was selected for further experiments as DMEC suggested the highest antiproliferative effect on U266 cells compared with the other two cell lines. In addition, DMEC (2, 5, 10, 20, 40, 80, and 160 μM) showed a low cell proliferation inhibition on normal spleen lymphocytes (<8%), indicating that DMEC (1-64 μM) had no obvious cytotoxic effects on plasma cells.

### Pro-apoptotic effect of DMEC on U266 cells

**Figure 3.**
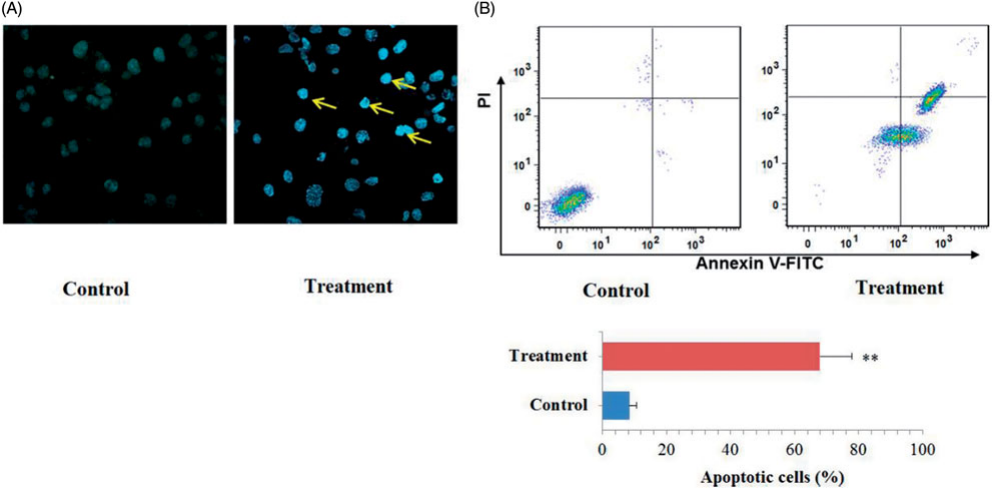
Pro-apoptotic effect of DMEC on U266 cells. (A) Hoechst 33324 staining assay. U266 cells were treated with DMEC (20 μM) for 24 h, stained with Hoechst 33324 and visualized under a fluorescent microscope (magnification, × 200). (B) Flow cytometry assay. U266 cells were treated with DMEC (20 μM) for 24 h, and then cells were stained with Annexin V-FITC/PI. Data are presented as the mean ± standard deviation (*n* = 4). **p < 0.01 *vs.* control group.

DMEC exerts obvious antiproliferative effect on the three MM cell lines according to the CCK-8 assay. To determine whether the antiproliferative effect of DMEC on MM cells was associated with an increase in apoptosis or not, U266 cells were stained with Hoechst 33324 to evaluate the changes in nuclear morphology. As shown in Figure 3(A), following treatment with 20 μM DMEC, obvious apoptosis features, including increased brightness, cell nuclear condensation, body shrinkage, as well as a decrease in the number of cells, was observed by fluorescent microscopy.

Furthermore, the pro-apoptotic effect of DMEC on U266 cells was evidenced by flow cytometry analysis. As displayed in Figure 3(B), the percentage of apoptotic cells was markedly increased after treatment with DMEC at concentration of 20 μM compared with the control (67.9 *vs.* 8.4%; *p* < 0.01). These results revealed that the antiproliferative effect of DMEC on human MM was associated with increased levels of apoptosis.

### DMEC induced mitochondria-mediated apoptosis on MM cells

**Figure 4.**
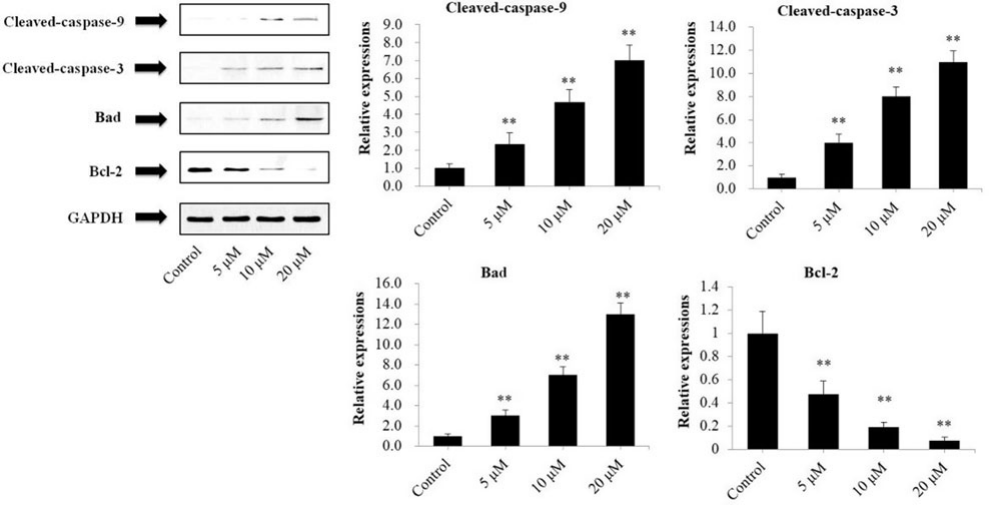
Exposure of U266 cells to DMEC resulted in upregulation of C-3, C-9 and Bad, and downregulation of Bcl-2. U266 cells were treated with DMEC (5, 10, and 20 μM) for 24 h and protein expression levels were determined by western blotting analysis using antibodies against C-3, C-9, Bad and Bcl-2. Data are presented as the mean ± standard deviation (*n* = 4). **p < 0.01 *vs.* control group.

**Figure 5.**
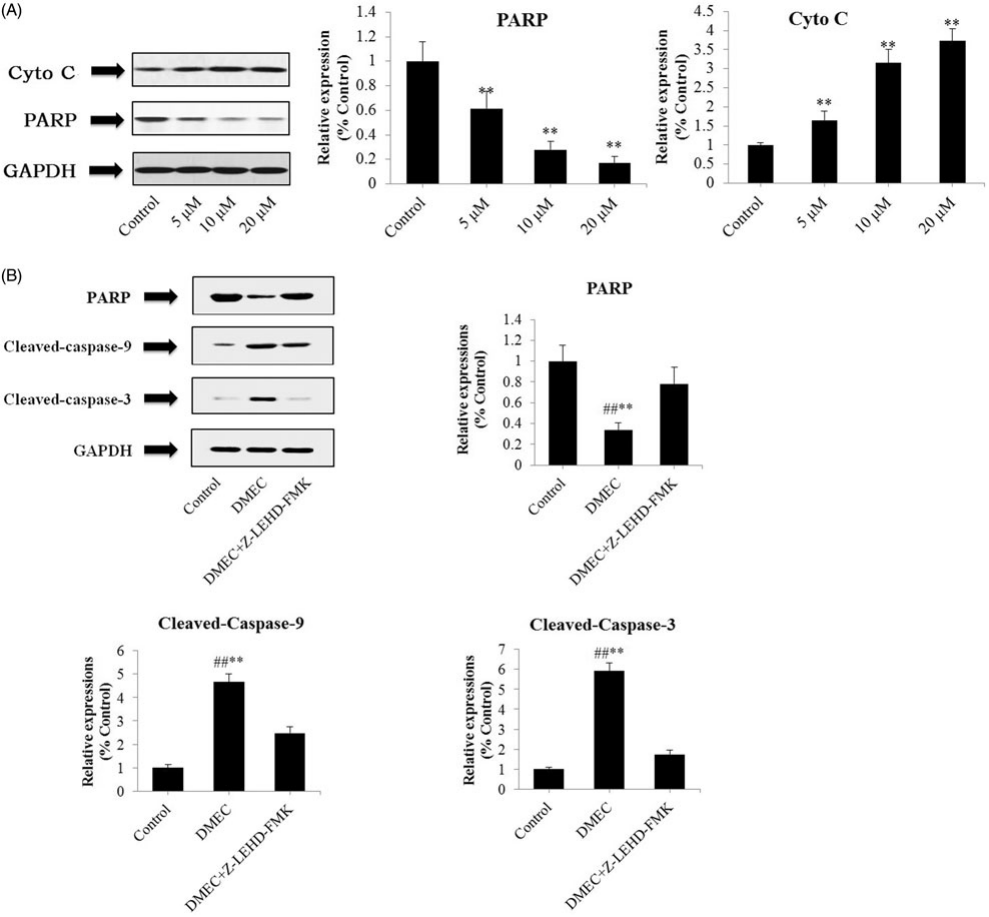
DMEC induced mitochondria-mediated apoptosis in MM cells (A) U266 cells were treated with DMEC (5, 10, and 20 μM) for 24 h, and protein expression levels of Cyto C and PARP were determined by western blot analysis. (B) U266 cells were treated with Z-LEHD-FMK (20 μM) for 1 h, followed by co-culture with 20 μM DMEC. Protein expression levels were determined by western blot analysis with the antibodies against PARP, C-3 and C-9. Data are presented as the mean ± standard deviation (*n* = 4). **p < 0.01 *vs.* control group; ^##^*p* < 0.01 *vs.* DMEC + Z-LEHD-FMK group.

Western blotting analysis was performed to investigate the related proteins associated with mitochondria-mediated intrinsic apoptosis in U266 cells, including C-3, C-9, Bad, Bcl-2, Cyto C and PARP. As exhibited in Figure 4, treatment with DMEC (5, 10, and 20 μM) markedly increased the protein expression levels of C-3 (*p* < 0.01), C-9 (*p* < 0.01), and Bad (*p* < 0.01), and decreased Bcl-2 expression (*p* < 0.01), in a concentration-dependent manner. As shown in Figure 5(A), Cyto C expression was obviously increased by DMEC treatment at 5, 10, and 20 μM (*p* < 0.01), whereas PARP expression was decreased (*p* < 0.01), especially at concentration of 20 μM.

To further assess whether the expression of PARP, C-3, and C-9 is essential for the apoptosis-promoting effect, U266 cells were treated with DMEC plus Z-LEHD-FMK (a C-9 inhibitor). As can be seen in Figure 5(B), DMEC (20 μM) treatment showed a significant downregulation of PARP expression (0.33 *±* 0.07), when compared with co-incubation of DMEC and Z-LEHD-FMK (0.78 *±* 0.16); however, C-3 and C-9 expression was obviously increased (5.91 *±* 0.41; 4.67 *±* 0.35). These findings indicated that the antiproliferative activity of DMEC against MM cells may be associated with inhibition of PARP, and activation of mitochondria-mediated apoptosis.

### Exposure of U266 cells to DMEC inhibited the PI3K/akt/mTOR signaling pathway

**Figure 6.**
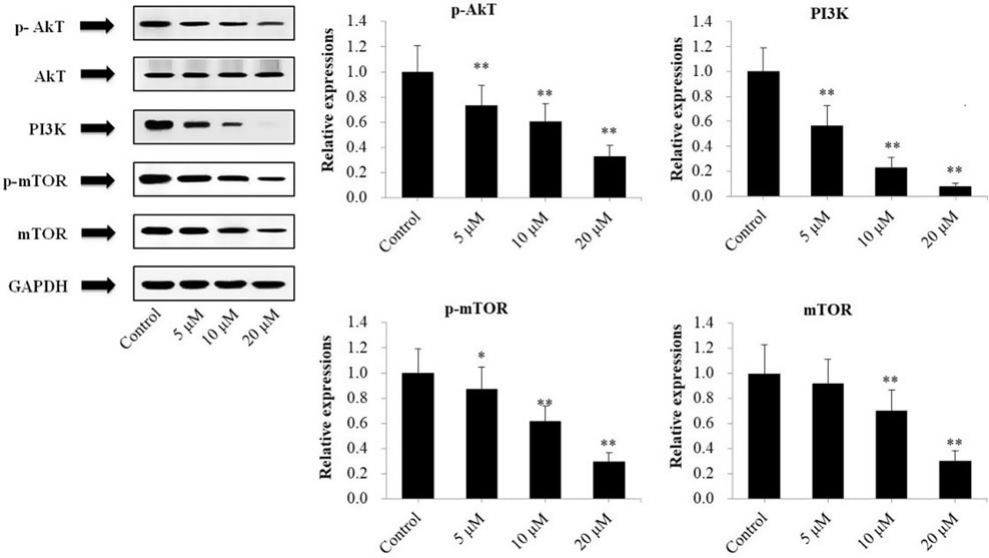
Exposure of U266 cells to DMEC inhibited the PI3K/Akt/mTOR signaling pathway. U266 cells were treated with DMEC (5, 10, and 20 μM) for 24 h, and protein expression levels were determined by western blotting analysis with antibodies against p-Akt, Akt, PI3K, p-mTOR and mTOR. Data are presented as the mean ± standard deviation (*n* = 4). *p < 0.05, **p < 0.01 *vs.* control group.

To further explore the mechanisms by which DMEC induced apoptosis in U266 cells, the PI3K/Akt/mTOR signaling pathway was evaluated. As shown in Figure 6, protein expression levels of PI3K (*p* < 0.01), p-Akt (*p* < 0.01), p-mTOR (*p* < 0.05 and *p* < 0.01) and mTOR (*p* < 0.01) in U266 cells were gradually decreased following exposure with increasing concentrations of DMEC (5, 10, and 20 μM) for 24 h.

### Pretreatment with insulin-like growth factor 1 (IGF-1) attenuated DMEC-induced apoptosis in U266 cells

**Figure 7.**
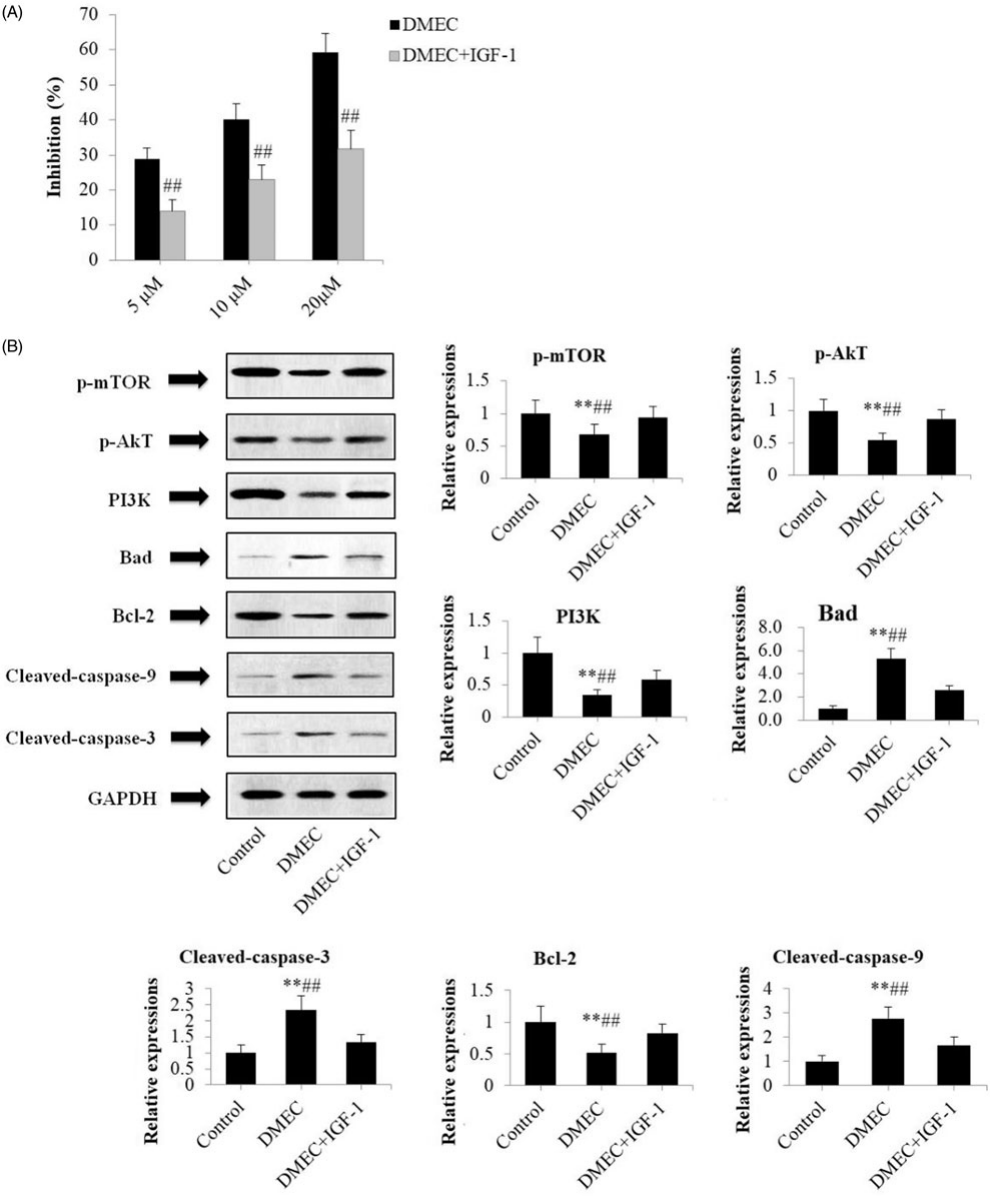
Pretreatment with IGF-1 decreased DMEC mediated apoptosis in U266 cells. (A) CCK-8 assay. U266 cells were treated with IGF-1 (100 ng/mL) for 1 h, followed by co-culture with DMEC (5, 10, and 20 μM) for 24 h. Subsequently, CCK-8 assay was used to determine the cell proliferation inhibition rate (*n* = 4). (B) Western blotting analysis. U266 cells were treated with IGF-1 (100 ng/mL) for 1 h, followed by co-culture with DMEC at 20 μM for 24 h. Protein expression levels were determined by western blotting analysis with the antibodies against p-mTOR, p-Akt, PI3K, Bad, Bcl-2, C-3 and C-9. Data are presented as the mean ± standard deviation (*n* = 4). **p < 0.01 *vs.* control group; ^##^*p* < 0.01 *vs.* DMEC + IGF-1 group.

To further confirm whether inhibition of the PI3K/Akt/mTOR signaling pathway is required for activation of DMEC-induced apoptosis, U266 cells were co-incubated with DMEC and IGF-1, a PI3K activator, for 24 h. It was identified that the antiproliferative effect of DMEC on U266 cells was obviously reduced by 14.92%, 16.97%, and 27.60% (Figure 7(A), *p* < 0.01), in a dose-dependent manner, when pretreated with 100 ng/mL IGF-1 followed by co-culture with DMEC (5, 10, and 20 μM). As shown in Figure 7(B), exposure of U266 cells to DMEC (20 μM) resulted in a significant increase in levels of C-3 (2.33 *±* 0.44), C-9 (2.78 *±* 0.48), and Bad (5.28 *±* 0.82), whereas Bcl-2 was decreased (0.51 *±* 0.13), compared with DMEC plus IGF-1 (1.33 *±* 0.24; 1.67 *±* 0.36; 2.57 *±* 0.41; 0.83 *±* 0.16). Furthermore, DMEC-induced decreased expression of PI3K (*p* < 0.01), p-Akt (*p* < 0.01), and p-mTOR (*p* < 0.01) were markedly reversed by pretreatment with IGF-1. Collectively, these findings further suggested that DMEC possessed an antiproliferative effect by promoting mitochondria-mediated intrinsic apoptosis *via* inhibition on the PI3K/Akt/mTOR signaling pathway.

## Discussion

To the best of our knowledge, this is the first report about the inhibitory effect of DMEC on MM cell lines. The results indicated that DMEC exhibited a notably antiproliferative effect against MM cell lines, including RPMI8226, MM.1S, and U266, by inducing mitochondria-mediated apoptosis *via* inhibition on the PI3K/Akt/mTOR signaling pathway.

CCK-8 assay, an effective alternative to the MTT assay (Li et al. 2016), was applied to evaluate the antiproliferative effect of DMEC against MM cells in this study. Apoptosis is central for sustaining the physiological equilibrium of cellular organisms (Zhang et al. 2016). It is characterized by various morphological and biochemical features, such as nuclear DNA fragmentation, rounding-up of the cell, decreased cell volume, apoptotic bodies, *etc.* (Fritz et al. 1984). Cancer cells are featured by abnormal cell proliferation, and thus, induction of apoptosis is considered as the main mechanism of many antitumor drugs (Fulda 2015; Heikaus et al. 2008; Obeid et al. 1993). The CCK-8 assay revealed that DMEC pretreatment dose-dependently inhibited the uncontrolled proliferation of MM cells lines RPMI8226, MM.1S, and U266 with IC_50_ of 25.97, 18.36, and 15.02 μM, respectively. Moreover, DMEC showed apoptotic effect on U266 cells (67.9 *vs.* 8.4%; *p* < 0.01), as indexed by increased brightness, nuclear condensation, and cell body shrinkage, and decreased cells number by fluorescent microscope and flow cytometer.

Mitochondria-mediated apoptosis has been regarded as an important apoptotic pathway, which mainly depends on the pro-apoptotic factors, such as C-3, C-9, and Bcl-2. Bcl-2 family proteins including Bad, Bax, and Bcl-2, the major regulators of apoptotic processes, play an essential role in mitochondria-mediated apoptosis (Brunelle and Letai 2009; Dai et al. 2017). It is generally considered that activation of Bcl-2 family proteins is the first regulatory step in mitochondria-mediated apoptosis. The interactions among Bcl-2 proteins family can regulate mitochondrial outer membrane permeabilization by release of Cyto C into cytoplasm, which plays a critical role in activating the inherent apoptosis pathway (Long et al. 2017; Vidya et al. 2010). The pro-apoptotic proteins, such as Bad, Bax, and Bcl-xs, can facilitate the release of Cyto C into the cytoplasm or suppress anti-apoptotic proteins, but Bcl-2 usually suppress the pro-apoptotic proteins of Bcl-2 (Jiang et al. 2014). Furthermore, caspases family proteins of C-3, caspase-8 and C-9, are largely responsible for apoptosis of tumor cells. C-9, the initiating caspase in a series caspase cascade reaction, can be activated by cytoplasmic Cyto C, further activates C-3, and then leads to a series of reactions that activate cell death proteases (Yang et al. 2004). The C-3 protein is regarded as an important biomarker for cells apoptosis (Peh et al. 2016). Treatment with DMEC increased the protein expression levels of C-3, C-9, Bad, and Cyto C, and decreased Bcl-2, indicating that the antiproliferative effect of DMEC is related to induction of mitochondria-mediated apoptosis, which was evidenced by co-incubation with DMEC and a C-9 inhibitor, Z-LEHD-FMK.

The PI3K/Akt/mTOR signaling pathway serves a central role in regulating normal cell physiology, cancer proliferation and tumorigenesis (Keppler-Noreuil et al. 2016). Increasing reports have revealed that inhibition on the PI3K/Akt/mTOR signaling pathway is critical for the antiproliferative effect on MM cells, as well as mediating resistance in therapeutics (Lkeda et al. 2010; Porta et al. 2014). Akt, also termed as threonine/serine kinase, is the main downstream target of PI3K (Du et al. 2015). Akt is activated and phosphorylated once the pleckstrin homology domain of Akt binds to PI3K-activated products, and Akt activation may inhibit pro-apoptotic factors of C-3, C-9, and Bad (Wu et al. 2018). The mTOR, belongs to the PI3K-associated serine/threonine kinase family, is a significant downstream target of Akt (Zhang et al. 2014). Dysregulation of PI3K/Akt/mTOR signaling pathway has been identified to be reflected in MM, and suppression of which is beneficial in prolonging progression-free survival and enhancing the life quality of patients with MM (Liu et al. 2009). The results suggested that DMEC inhibited expression of PI3K, Akt, and mTOR in MM cells, which was evidenced by pretreatment with IGF-1 (stimulator of cancer cell proliferation) in U266 cells. The current result was consistent with a previous study (Vijay and Shaji 2018) indicating that the PI3K/Akt/mTOR pathway is critical for the apoptosis of MM cells and may be considered a promising and feasible therapeutic target for MM.

## Conclusions

The current study indicates that DMEC possesses notable antiproliferative effect on human MM cell lines, and its potential molecular mechanisms may be associated with mitochondria-mediated apoptosis *via* inhibition on the PI3K/Akt/mTOR signaling pathway. Thus, DMEC is a promising candidate for the treatment of MM. However, an *in vivo* animal experiment in a relevant multiple myeloma model is still strongly required. This also provides a new insight into the pharmacological effect of DMEC from *Caragana pruinosa,* which would be beneficial for the development of this plant.
